# Immunization coverage, knowledge, satisfaction, and associated factors of non-National Immunization Program vaccines among migrant and left-behind families in China: evidence from Zhejiang and Henan provinces

**DOI:** 10.1186/s40249-023-01145-5

**Published:** 2023-10-13

**Authors:** Yaguan Zhou, Duanhui Li, Yuan Cao, Fenhua Lai, Yu Wang, Qian Long, Zifan Zhang, Chuanbo An, Xiaolin Xu

**Affiliations:** 1 School of Public Health, The Second Affiliated Hospital, Zhejiang University School of Medicine, Yuhangtang Road 866, Hangzhou, 310058 Zhejiang China; 2 The Key Laboratory of Intelligent Preventive Medicine of Zhejiang Province, Hangzhou, Zhejiang China; 3 Songxian Center for Disease Control and Prevention, Luoyang, Henan China; 4 Department of Immunoprophylaxis, Xiaoshan Center for Disease Control and Prevention, Hangzhou, Zhejiang China; 5 Global Health Research Center, Division of Social Sciences, Duke Kunshan University, Kunshan, Jiangsu China; 6 Global Health Research Center, Duke Kunshan University, Kunshan, Jiangsu China; 7 School of Public Health, Faculty of Medicine, The University of Queensland, Brisbane, Australia

**Keywords:** Non-National Immunization Program vaccines, Migrant family, Left-behind family, Immunization coverage, Immunization knowledge, Immunization satisfaction, China

## Abstract

**Background:**

Migrant and left-behind families are vulnerable in health services utilization, but little is known about their disparities in immunization of non-National Immunization Program (NIP) vaccines. This study aims to evaluate the immunization coverage, knowledge, satisfaction, and associated factors of non-NIP vaccines among local and migrant families in the urban areas and non-left-behind and left-behind families in the rural areas of China.

**Methods:**

A cross-sectional survey was conducted in urban areas of Zhejiang and rural areas of Henan in China. A total of 1648 caregivers of children aged 1–6 years were interviewed face-to-face by a pre-designed online questionnaire, and their families were grouped into four types: local urban, migrant, non-left-behind, and left-behind. Non-NIP vaccines included *Hemophilus influenza* b (Hib) vaccine, varicella vaccine, rotavirus vaccine, enterovirus 71 vaccine (EV71) and 13-valent pneumonia vaccine (PCV13). Log-binomial regression models were used to calculate prevalence ratios (*PR*s) and 95% confidence intervals (*CI*s) for the difference on immunization coverage of children, and knowledge and satisfaction of caregivers among families. The network models were conducted to explore the interplay of immunization coverage, knowledge, and satisfaction. Logistic regression models with odds ratios (*OR*s) and 95% *CI*s were used to estimate the associated factors of non-NIP vaccination.

**Results:**

The immunization coverage of all non-NIP vaccines and knowledge of all items of local urban families was the highest, followed by migrant, non-left-behind and left-behind families. Compared with local urban children, the *PR*s (95% *CI*s) for getting all vaccinated were 0.65 (0.52–0.81), 0.29 (0.22–0.37) and 0.14 (0.09–0.21) among migrant children, non-left-behind children and left-behind children, respectively. The coverage-knowledge-satisfaction network model showed the core node was the satisfaction of vaccination schedule. Non-NIP vaccination was associated with characteristics of both children and caregivers, including age of children (> 2 years-*OR*: 1.69, 95% *CI*: 1.07–2.68 for local urban children; 2.67, 1.39–5.13 for migrant children; 3.09, 1.23–7.76 for non-left-behind children); and below caregivers’ characteristics: family role (parents: 0.37, 0.14–0.99 for non-left-behind children), age (≤ 35 years: 7.27, 1.39–37.94 for non-left-behind children), sex (female: 0.49, 0.30–0.81 for local urban children; 0.31, 0.15–0.62 for non-left-behind children), physical health (more than average: 1.58, 1.07–2.35 for local urban children) and non-NIP vaccines knowledge (good: 0.45, 0.30–0.68 for local urban children; 7.54, 2.64–21.50 for left-behind children).

**Conclusions:**

There were immunization disparities in non-NIP vaccines among migrant and left-behind families compared with their local counterparts. Non-NIP vaccination promotion strategies, including education on caregivers, and optimization of the immunization information system, should be delivered particularly among left-behind and migrant families.

**Graphical Abstract:**

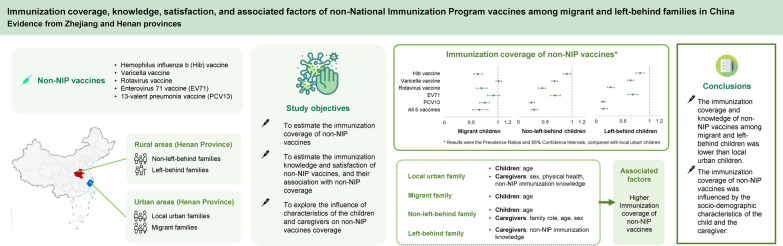

**Supplementary Information:**

The online version contains supplementary material available at 10.1186/s40249-023-01145-5.

## Background

With the development of economic and urbanization, a large proportion of people move from rural to urban areas in order to get a better life in the context of national labor shortages in China over the past few decades [1, 2]. The mass migration of laborers caused a growth of two special groups: left-behind families in rural areas and migrant families in urban areas [3–5]. Left-behind families refer to one or both parents migrated into cities for work, leaving their children in the rural communities with other caregivers (e.g., grandparents) for over six months [6]; while migrant families refer to parents migrated into cities for work together with their children for over six months [7]. Till 2022, there was an estimated 6.44 million left-behind children and over 70 million migrant children in China [8]. According to the household registration policy in China, “hukou” gives households access to social benefits in their registration areas but limits access to those outside their registration area, which causes that migrant families often have less access to social resources, including housing, education and medical services [9]. On the other hand, left-behind families in rural areas, characterized by parent–child separation [10], were also socially disadvantaged. Therefore, compared to children of local families, those of migrant and left-behind families usually experienced poorer health, lower quality of life, and lower utilization of health services [11–13]. The widening health inequality in health services use (e.g., immunization) in migrant and left-behind children has become an important issue in China [9].

Immunization has been considered as one of the most successful strategies for prevention and control of infectious diseases. Since the National Immunization Program (NIP) was implemented in 1978, China has been working to promote childhood immunization and reduce national burden of vaccine-preventable diseases among children [14]. In China, vaccines were divided into two categories: NIP and non-NIP vaccines [15]. Non-NIP vaccines, an alternative and supplement to NIP vaccines, also contribute to reduce morbidity of some essential infectious diseases (e.g., hepatitis A, and influenza) [16]. Unlike NIP vaccines to be free and mandatory, non-NIP vaccines are voluntarily administered and self-funded, therefore families usually show inadequate knowledge level and low acceptance of non-NIP vaccination for their children, which even widens disparities of immunization among migrant and left-behind families [15, 17].

Early findings have assessed the disparities in immunization between migrant or left-behind children and local children. A systematic review and meta-analysis of 11 studies found rural–urban migrant children in China, India and Nigeria had lower immunization coverage of NIP vaccines than local urban children [2]. A cross-sectional study conducted in Guangdong Province of China also found the immunization coverage of NIP vaccines among migrant children aged 12–59 months was quite low, influenced by the sex and birth place of children, and the caregivers’ occupation, knowledge and attitude towards immunization, as well as the family income [18]. For left-behind children, one study suggested their age-appropriate vaccination coverage for NIP vaccines was significantly lower than that in non-left-behind children [19]. Moreover, the association of immunization coverage, knowledge, practice, and experiences has been reported in several studies. For example, a study among 1820 migrant children aged 12–35 months in Beijing of China suggested that the age-appropriate immunization coverage was significantly influenced by the caregivers’ immunization knowledge but not the satisfaction with vaccination services [20]. Findings from guardians of 0–59-month-old Chinese children reported the coverage of influenza vaccines was negatively related to hesitancy, while vaccine hesitancy could decreased with knowledge and vaccination experience improved [21]. However, previous studies have several limitations: (1) mostly focused on NIP vaccines, (2) only focused on one or a few types of non-NIP vaccines or did not specify the category of vaccines, and (3) did not include children from both the left-behind and migrant families and their local counterparts in one study design. To narrow or eliminate the immunization disparities among migrant and left-behind children, it is necessary to evaluate the immunization coverage, knowledge and satisfaction and their interplays among migrant and left-behind families, particularly in one study design.

Using information from migrant and left-behind families and their local counterparts in China, this study was conducted (1) to estimate the immunization coverage of non-NIP vaccines; (2) to estimate the immunization knowledge and satisfaction of non-NIP vaccines, and their association with non-NIP coverage; and (3) to explore the influence of characteristics of the children and caregivers on non-NIP vaccines coverage.

## Methods

### Study design and data collection

A cross-sectional survey was conducted during July and October 2022 in Zhejiang and Henan Provinces respectively, either of which is the major labor-importing or labor-exporting province in China and exists a large number of migrant families or left-behind families. The target population of this survey was made up of caregivers of children aged 1–6 years, who resided in urban areas of Xiaoshan District, Hangzhou City, Zhejiang Province and rural areas of Song County, Luoyang City, Henan Province. Families of caregivers included in Xiaoshan district were migrant families and local urban families, and those of caregivers included in Song County were left-behind families and non-left-behind families. Caregivers were excluded if (1) they have been residing locally for < 6 months; or (2) their children could not be vaccinated because of severe illness or disabilities.

Five towns/communities were selected in Song County and Xiaoshan District using the simple random sampling method, and caregivers were recruited in local township health centers and community health service centers. By reviewing the immunization coverage of non-NIP vaccines among urban and rural children (75.8%), using a desired precision of ± 2% with 95% confidence intervals, and assuming a non-response rate of 10.0%, the required number of surveyed caregivers of children was 1937.

The questionnaire included five sections (characteristics of children, characteristics of caregivers, immunization coverage of children, immunization knowledge of caregivers, and immunization satisfaction of caregivers) and 51 questions. The questionnaire was administered on the online platform Wenjuanxing (https://www.wjx.cn/), the most popular web-based questionnaire platform in China. There were standard instructions for the questionnaire survey, and a total of 35 primary care physicians were invited to administrate the questionnaires. It would take about 10 min to complete the questionnaire, and the caregivers were incentivized to participate in the survey by providing reasonable money. In our survey, only one caregiver per child was face-to-face interviewed, whose answers were entered real-time to the Wenjuanxing platform by primary care physicians. Before the survey, each caregiver of the child enrolled was asked to provide electronic informed consent on the Wenjuanxing online platform. Zhejiang University School of Public Health Medicine Ethics Committees approved the study protocol (ZGL202206-6).

### Immunization coverage, knowledge, satisfaction

The five types of non-NIP vaccines included in our survey were *Haemophiles influenza* b (Hib) vaccine, varicella vaccine, rotavirus vaccine, enterovirus 71 vaccine (EV71) and 13-valent pneumonia vaccine (PCV13). These five vaccines were commonly used in China, but have not been included in the NIP [22]. Caregivers were asked whether their children had been vaccinated the aforementioned non-NIP vaccines, and the coverage of non-NIP vaccines was calculated among local urban, migrant, non-left-behind and left-behind children respectively.

The immunization knowledge of caregivers was measured using seven items, including convenience, category, efficiency, continuity, time, schedule, and adverse events of non-NIP vaccines, which were calculated among local urban, migrant, non-left-behind and left-behind families respectively. The description of each item was provided to caregivers, and they were asked whether they aware of these seven items. For example, the knowledge item of “convenience” was measured by asking caregivers whether they were aware of that children could accept non-NIP vaccination nationwide if they had a vaccination certificate.

The immunization satisfaction of caregivers was investigated using nine items, including convenience, vaccination reminder, vaccination environment, consultation, vaccination skills, service quality, vaccination process, vaccination education and time of vaccination, which were calculated among local urban, migrant, non-left-behind and left-behind families respectively. Similarly, a description of each item was provided to caregivers, and caregivers were asked whether they were satisfied with the aforementioned nine items. For example, the experience item of “schedule” was measured by asking caregivers whether they were satisfied with the non-NIP vaccination schedule of local clinics.

Questions for immunization knowledge and satisfaction were general to all non-NIP vaccines, and the whole questionnaire was presented in the Additional file 1: Questionnaire.

### Characteristics of families

The characteristics of children included age, sex (male; female) and birth order (first-born; later-born). The characteristics of caregivers included family role (parents; others), age (≤ 35 years; 35–40 years; ≥ 40 years), sex (male; female), education level (elementary school or lower; middle school; junior college or higher), total household income (less than average; more than average), physical health [assessed by 12-Item Short Form Survey (SF-12)] and mental health (assessed by SF-12). Total household income was measured by the sum of earning income, capital income, pension income, income from government transfers, other income and the total income from other household members during last year.

### Statistical analysis

Characteristics of families were described as mean [standard deviation (SD)] for continuous variables and as number (percentage) for categorical variables. The differences of variables across groups by family types were compared using one-way analysis of variance (ANOVA) and Chi-square test.

Log-binomial regression models were used to calculate prevalence ratios (*PR*s) and 95% confidence intervals (*CI*s) for the difference on immunization coverage of children, and knowledge and satisfaction of caregivers of different family types, regarding local urban families as the reference. Age and sex of children were adjusted in the log-binomial regression models. The coverage-knowledge-satisfaction network models of non-NIP vaccination were conducted through Mixed Graphical Model (MGM) using the R package “qgraph” [23]. Least Absolute Shrinkage and Selection Operator (LASSO) and Extended Bayesian Information Criterion (EBIC) were utilized to reduce the pseudo-correlation of connections in the network and to find the optimal fitting model [24]. The nodes of the network represent different items in coverage, knowledge, and satisfaction of non-NIP vaccines, while the bridges represent the correlation between nodes, which are represented by calculating the coefficient of partial correlation among the items of vaccines coverage, vaccination knowledge and satisfaction. The closer the nodes are and the thicker the connected line segments are, the stronger the correlations between nodes are. Strength, defined as the absolute value of the shortest distance between all connections of a node, was chosen to represent the centrality of each node, and its magnitude could represent the extent of connection between nodes in the network. Bridge expected influence refers to the sum of the absolute value of the shortest distance between a specific node and all other connected nodes, which was proportionable to the degree of network conduction through the node [25]. In addition, we used stability coefficient (CS) and 95% *CI* of edge weights to evaluate the stability of the network. CS coefficient represents the maximum sample attenuation ratio when the correlation value between the original network and the regenerative network parameters is 0.70 [26].

The multivariable logistic regression models to conducted to explore the associated factors of non-NIP vaccination among four types of families, with the outcomes of non-NIP vaccination divided into two groups: “having received all five non-NIP vaccines” and “having not received at least one non-NIP vaccines”. Odds ratios (*OR*s) and 95% *CI*s were calculated to compare the association of the characteristics of children and caregivers with non-NIP vaccination for each type of families.

Analyses were performed using SAS (Version 9.4, SAS Institute Inc., Cary, NC) and R (Version 3.6.1; R studio, Boston, Massachusetts). The study was conducted and reported in line with the Consensus-Based Checklist for Reporting of Survey Studies (CROSS, Additional file 2: Table).

## Results

### Basic characteristics

A total of 2366 caregivers were invited to participate in the survey, 2031 agreed to start, and 2018 completed the survey, resulting in response rate of 85.3% and completion rate of 99.4%. Considering the first shot of included non-NIP vaccines were scheduled before one year old, we further excluded respondents with children less than one year old to avoid underestimating the immunization of coverage non-NIP vaccines. Therefore, a number of 1648 caregivers of children aged 1–6 years were included in the analyses. The average age of children was 3.45 (± 1.59) years, and 47.9% of the children were females. The characteristics of four family types are presented in Table 1. The number of local urban children, migrant children, non-left-behind children and left-behind children was 517 (31.4%), 276 (16.8%), 488 (29.6%) and 367 (22.3%), respectively. Migrant children are more likely to be younger and first-born, and their caregivers are more likely to be parents, 35 years or younger and females, and have lower household income. Left-behind children are more likely to be older and first-born, and their caregivers are more likely to be other than parents, ≥ 40 years old and males, and have lower education level and higher household income.

**Table 1:** Basic characteristics of children and the caregivers according to four types of family in urban areas in Zhejiang Province and rural areas in Henan Province, China 2022 (*n* = 1648)

	Total (*N* = 1648)	Urban		Rural		*χ*^2^/F	*P* value
		Local (*n* = 517)	Migrant (*n* = 276)	Non-left-behind (*n* = 488)	Left-behind (*n* = 367)		
Children							
Age, years	3.45 (1.59)	3.59 (1.71)	3.00 (1.62)	3.44 (1.50)	3.58 (1.44)	9.66	< 0.0001
Sex							
Male	859 (52.1%)	281 (54.4%)	144 (52.2%)	254 (52.1%)	180 (49.1%)	2.42	0.4894
Female	789 (47.9%)	236 (45.6%)	132 (47.8%)	234 (47.9%)	187 (50.9%)		
Birth order							
First-born	671 (40.8%)	198 (38.4%)	122 (44.2%)	176 (36.1%)	175 (47.7%)	14.08	0.0028
Later-born	974 (59.2%)	317 (61.6%)	154 (55.8%)	311 (63.9%)	192 (52.3%)		
Caregivers							
Family role							
Parents	1112 (67.5%)	320 (61.9%)	238 (86.2%)	438 (89.8%)	116 (31.6%)	377.09	< 0.0001
Others	536 (32.5%)	197 (38.1%)	38 (13.8%)	50 (10.2%)	251 (68.4%)		
Age, years							
≤ 35	1056 (64.1%)	355 (68.6%)	235 (85.1%)	362 (74.2%)	104 (28.3%)	565.09	< 0.0001
35–40	212 (12.9%)	98 (19.0%)	30 (10.9%)	71 (14.5%)	13 (3.6%)		
≥ 40	380 (23.0%)	64 (12.4%)	11 (4.00%)	55 (11.3%)	250 (68.1%)		
Sex							
Male	287 (17.4%)	89 (17.2%)	37 (13.4%)	72 (14.8%)	89 (24.2%)	17.43	0.0006
Female	1361 (82.6%)	428 (82.8%)	239 (86.6%)	416 (85.2%)	278 (75.8%)		
Education level							
Elementary school or lower	255 (15.6%)	5 (1.0%)	8 (2.9%)	54 (11.1%)	188 (51.2%)	1225.66	< 0.0001
Middle school	863 (52.5%)	91 (17.6%)	201 (72.8%)	398 (81.6%)	173 (47.1%)		
Junior college or higher	530 (32.2%)	421 (81.4%)	67 (24.3%)	36 (7.4%)	6 (1.6%)		
Total household income							
Less than average	977 (59.3%)	331 (64.0%)	193 (69.9%)	252 (51.6%)	197 (53.7%)	31.96	< 0.0001
More than average	671 (40.7%)	186 (36.0%)	83 (30.1%)	236 (48.4%)	170 (46.3%)		
Physical health score	53.60 (6.55)	54.05 (5.95)	53.40 (5.24)	54.45 (6.26)	51.99 (8.14)	11.24	< 0.0001
Mental health score	56.72 (8.93)	55.90 (9.02)	58.80 (7.82)	56.16 (9.23)	57.05 (8.94)	7.31	< 0.0001

The age of children, and the physical health and mental health score of caregivers were presented using mean and standard deviations. Other variables were presented using number (percentage). The average annual total household income was 300,000 Chinese Yuan (CNY), 200,000 CNY, 40,000 CNY and 40,000 CNY for local urban children, migrant children, non-left-behind children and left-behind children, local urban children and migrant children, respectively

### Immunization coverage, knowledge, and satisfaction

#### Immunization coverage

**Fig. 1:**
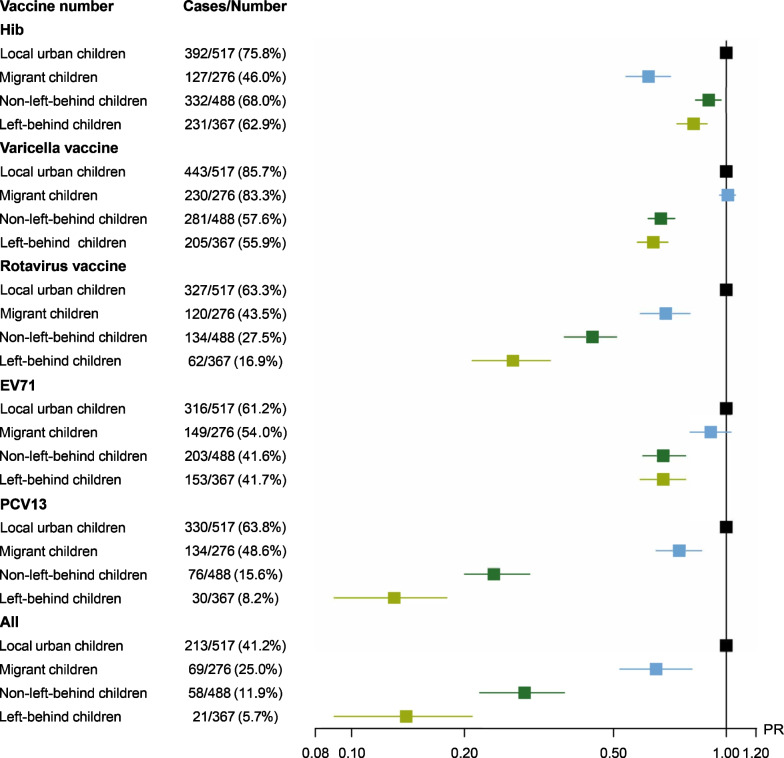
The immunization coverage, prevalence ratio (*PR*s) and 95% confidence intervals (*CI*s) of non-National Immunization Program vaccines among children from four types of family in urban areas in Zhejiang Province and rural areas in Henan Province, China 2022. *Hib Hemophilus influenza* b; *EV71* enterovirus 71 vaccine, *PCV13* 13-valent pneumonia vaccine; *PR* prevalence ratio

The immunization coverage of non-NIP vaccines among children of different family types is presented in Fig. 1. The coverage for single non-NIP vaccine was highest among local urban children, ranging from 61.1% for EV71 to 85.7% for varicella vaccine. The coverage for varicella vaccine, rotavirus vaccines, EV71 and PCV13 were lowest among left-behind children, which were 55.9%, 16.9%, 41.7%, and 8.2%, respectively. The coverage of all five non-NIP vaccines was highest among local urban children (41.2%), and was lowest among left-behind children (5.7%). After adjustment for age and sex of children, the *PR*s (95% *CI*s) for getting all vaccinated were 0.65 (0.52–0.81), 0.29 (0.22–0.37) and 0.14 (0.09–0.21) among migrant children, non-left-behind children and left-behind children, respectively.

#### Immunization knowledge

Table 2 shows the immunization knowledge of non-NIP vaccines among the caregivers of different family types. Among the seven knowledge items of non-NIP vaccination, vaccination convenience, category, time and schedule were most aware by the caregivers, while the knowledge of vaccination continuity was lowest. For example, the awareness rate of vaccination time and schedule was 99.2% and 98.9% respectively, while that of vaccination continuity was 27.3% among caregivers from left-behind families. The rate of being aware of all knowledge items was highest in local urban families (41.2%), followed by migrant (23.2%), non-left-behind (19.5%) and left-behind families (11.4%). Compared to caregivers from local urban families, the *PR*s (95% *CI*s) for being aware of all knowledge items were 0.55 (0.43–0.70), 0.47 (0.38–0.58) and 0.28 (0.21–0.38) among those from migrant, non-left-behind and left-behind families, respectively.

**Table 2:** Awareness rate, prevalence ratios (*PR*s) and 95% confidence intervals (*CI*s) of immunization knowledge of non-NIP vaccines among the caregivers from four family types in urban areas in Zhejiang Province and rural areas in Henan Province, China 2022 (*n* = 1648)

	Local urban (*n* = 517)	Migrant (*n* = 276)	Non-left-behind (*n* = 488)	Left-behind (*n* = 367)
Awareness, *n* (%)				
Vaccine convenience	487 (94.2)	268 (97.1)	472 (96.7)	338 (92.1)
Vaccine category	494 (95.6)	255 (92.4)	480 (98.4)	354 (94.5)
Vaccine efficiency	490 (94.8)	249 (90.2)	472 (96.7)	334 (91.0)
Vaccination continuity	300 (58.0)	135 (48.9)	165 (33.8)	100 (27.3)
Vaccination time	488 (94.4)	268 (97.1)	482 (98.8)	364 (99.2)
Vaccination schedule	497 (96.1)	265 (96.0)	473 (96.9)	363 (98.9)
Adverse event	427 (82.6)	165 (59.8)	398 (81.6)	271 (73.8)
All	213 (41.2)	64 (23.2)	95 (19.5)	42 (11.4)
*PR* (95% *CI*)				
All	1.00	0.55 (0.43–0.70)	0.47 (0.38–0.58)	0.28 (0.21–0.38)

*PR* prevalence ratio; *CI* confidence interval, *NIP* National Immunization Program

#### Immunization satisfaction

Table 3 shows the immunization satisfaction of non-NIP vaccines among the caregivers of different family types. More than 90% of the caregivers reported satisfied with the nine items of non-NIP vaccination satisfaction. The satisfaction rate was 90.5% (vaccination reminder)–94.6% (vaccination skills), 91.3% (vaccination education)–96.0% (vaccination skills), 90.4% (convenience)–96.1% (vaccination skills), and 95.1% (convenience)–99.2% (vaccination reminder) among those from local urban, migrant, non-left-behind families and left-behind families. Compared to caregivers from local urban families, caregivers from left-behind families had higher satisfaction rate of non-NIP vaccination (*PR*: 1.12, 1.05–1.19 for being satisfied with all items), while the PRs for being satisfied with all items were not significant among those from migrant and non-left-behind families.

**Table 3:** Satisfaction rate, prevalence ratios (*PR*s) and 95% confidence intervals (*CI*s) of non-NIP vaccination among the caregivers from four family types in urban areas in Zhejiang Province and rural areas in Henan Province, China 2022 (*n* = 1648)

	Local urban (*n* = 517)	Migrant (*n* = 276)	Non-left-behind (*n* = 488)	Left-behind (*n* = 367)
Satisfaction, *n* (%)				
Convenience	479 (92.7)	254 (92.0)	441 (90.4)	349 (95.1)
Vaccination reminder	468 (90.5)	257 (93.1)	466 (95.5)	364 (99.2)
Vaccination environment	481 (93.0)	257 (93.1)	453 (92.8)	357 (97.3)
Consultation	475 (91.9)	261 (94.6)	453 (92.8)	360 (98.1)
Vaccination skills	489 (94.6)	265 (96.0)	469 (96.1)	363 (98.9)
Service quality	481 (93.0)	261 (94.6)	459 (94.1)	359 (97.8)
Vaccination process	481 (93.0)	258 (93.5)	464 (95.1)	362 (98.6)
Vaccination education	477 (92.3)	252 (91.3)	461 (94.5)	359 (97.8)
Time of vaccination	480 (92.8)	254 (92.0)	464 (95.1)	362 (98.6)
All items	427 (82.6)	221 (80.1)	419 (85.9)	343 (93.5)
*PR* (95% *CI*)				
All items	1.00	0.97 (0.90–1.04)	1.04 (0.99–1.10)	1.12 (1.05–1.19)

*PR* prevalence ratio, *CI* confidence interval, *NIP* National Immunization Program

#### The interrelationship among immunization coverage, knowledge and satisfaction

**Fig. 2:**
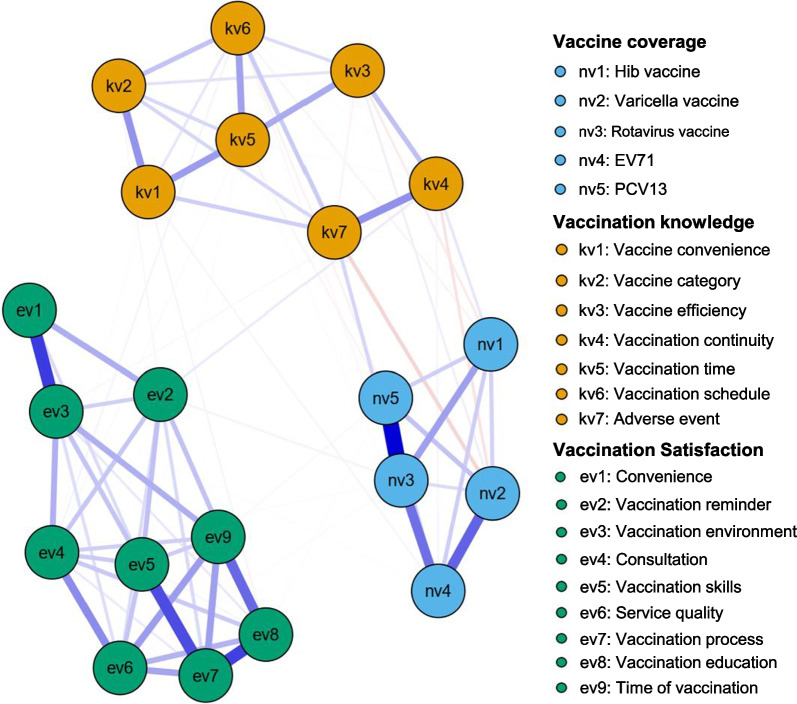
Coverage-knowledge-satisfaction network model of non-NIP vaccination among included families in urban areas in Zhejiang Province and rural areas in Henan Province, China 2022. *Hib Hemophilus influenza* b; *EV71* enterovirus 71 vaccine, *PCV13* 13-valent pneumonia vaccine

Figure 2 displays the coverage-knowledge-satisfaction network model of non-NIP vaccination for children aged 1–6 years. In the types of vaccines, the connection between rotavirus vaccine and PCV13 was strongest, with the edge weight of 0.47. In the knowledge items of non-NIP vaccination, the connection between convenience and vaccines category, as well as the connection between vaccination continuity and adverse events were strongest, with the edge weights of 0.20. In the immunization satisfaction items of non-NIP vaccines, the connection between convenience and vaccination environment was strongest (edge weight: 0.36), followed by the connection between vaccination schedule and vaccination education (edge weight: 0.35). The core nodes in the network models includes vaccination schedule, rotavirus vaccine, and vaccination education due to their high strength. The primary bridge between non-NIP vaccines coverage and knowledge was the rotavirus vaccine node, and the second ones were vaccination continuity and vaccine category.

### Associated factors of non-NIP vaccination

Table 4 shows the association of characteristics of families with immunization coverage of non-NIP vaccines across four family types. Children aged > 2 years among local urban (*OR* = 1.69, 95% *CI*: 1.07–2.68), migrant (2.67, 1.39–5.13) and non-left-behind families (3.09, 1.23–7.76) were more likely to get all vaccines. Better physical health of caregivers from local urban families was associated with an increased odds (1.58, 1.07–2.35) of receiving all non-NIP vaccines for their children, while female caregivers (0.49, 0.30–0.81), and caregivers with better immunization knowledge (0.45, 0.30–0.68) showed lower probability to have their children vaccinated all non-NIP vaccines. Among non-left-behind families, younger caregivers were more likely to get their children vaccinated (7.27, 1.39–37.94), while those being parents (0.37, 0.14–0.99) or females (0.31, 0.15–0.62) were less likely to do so. As for left-behind families, good immunization knowledge of caregivers increased their children's odds of have all vaccinated (7.54, 2.64–21.50).

**Table 4:** Odds ratios (*OR*s) and 95% confidence interval (*CI*s) for the association of characteristics of families with non-NIP vaccine coverage among four types of family in urban areas in Zhejiang Province and rural areas in Henan Province, China 2022 (*n* = 1648)

	Local urban (*n* = 517)		Migrant (*n* = 276)		Non-left-behind (*n* = 488)		Left-behind (*n* = 367)	
	*OR* (95% *CI*)	*P* value	*OR* (95% *CI*)	*P* value	*OR* (95% *CI*)	*P* value	OR (95% *CI*)	*P* value
**Children**								
Age, years								
1–2	Ref.		Ref.		Ref.		Ref.	
> 2	**1.69 (1.07–2.68)**	**0.025**	**2.67 (1.39–5.13)**	**0.003**	**3.09 (1.23–7.76)**	**0.017**	1.83 (0.44–7.55)	0.406
Sex								
Male	0.93 (0.64–1.36)	0.707	0.83 (0.46–1.50)	0.542	1.40 (0.76–2.58)	0.277	1.54 (0.58–4.11)	0.389
Female	Ref.		Ref.		Ref.		Ref.	
Birth order								
First-born	1.21 (0.81–1.79)	0.357	0.86 (0.47–1.58)	0.629	1.02 (0.55–1.87)	0.970	0.85 (0.32–2.25)	0.750
Later-born	Ref.		Ref.		Ref.		Ref.	
**Caregivers**								
Family role								
Parents	0.86 (0.58–1.27)	0.437	1.65 (0.68–4.03)	0.272	**0.37 (0.14–0.99)**	**0.047**	0.51 (0.11–2.46)	0.404
Others	Ref.		Ref.		Ref.		Ref.	
Age, years								
≤ 35	0.71 (0.39–1.29)	0.257	0.74 (0.17–3.27)	0.689	**7.27 (1.39–37.94)**	**0.019**	1.01 (0.21–5.01)	0.987
35–40	0.60 (0.30–1.19)	0.145	0.87 (0.17–4.54)	0.864	3.88 (0.67–22.57)	0.131	–	0.982
≥ 40	Ref.		Ref.		Ref.		Ref.	
Sex								
Male	Ref		Ref		Ref		Ref	
Female	**0.49 (0.30–0.81)**	**0.005**	0.81 (0.35–1.87)	0.616	**0.31 (0.15–0.62)**	**0.001**	2.80 (0.68–11.62)	0.155
Education level								
Elementary school or lower	Ref.		Ref.		Ref.		Ref.	
Middle school	–	0.951	1.37 (0.24–7.91)	0.723	1.33 (0.41–4.34)	0.641	1.24 (0.37–4.17)	0.731
Junior college or higher	–	0.951	2.30 (0.37–14.23)	0.370	0.77 (0.13–4.46)	0.773	–	0.988
Total household income								
Less than average	Ref.		Ref.		Ref.		Ref.	
More than average	1.35 (0.91–2.01)	0.139	1.11 (0.56–2.23)	0.763	1.08 (0.59–1.97)	0.810	1.91 (0.63–5.76)	0.253
Physical health score								
Less than average	Ref.		Ref.		Ref.		Ref.	
More than average	**1.58 (1.07–2.35)**	**0.023**	1.37 (0.74–2.55)	0.321	1.14 (0.59–2.20)	0.695	2.38 (0.72–7.86)	0.154
Mental health score								
Less than average	Ref.		Ref.		Ref.		Ref.	
More than average	0.71 (0.47–1.06)	0.097	0.56 (0.29–1.07)	0.078	0.57 (0.31–1.03)	0.064	0.48 (0.18–1.30)	0.150
Knowledge of non-NIP vaccination								
Poor	Ref.		Ref.		Ref.		Ref.	
Good	**0.45 (0.30–0.68)**	**< 0.0001**	0.57 (0.27–1.18)	0.130	0.82 (0.37–1.83)	0.626	**7.54 (2.64–21.50)**	**< 0.001**
Satisfaction of non-NIP vaccination								
Not satisfied	Ref.		Ref.		Ref.		Ref.	
Satisfied	0.80 (0.48–1.31)	0.368	0.60 (0.30–1.20)	0.150	0.56 (0.26–1.18)	0.128	0.25 (0.06–1.10)	0.068

Bold values indicate statistical significance. The average annual total household income was 300,000 Chinese Yuan (CNY), 200,000 CNY, 40,000 CNY, and 40,000 CNY for local urban children, migrant children, non-left-behind children and left-behind children, respectively

*OR* odds ratio, *CI* confidence interval, *NIP* National Immunization Program

## Discussion

Through this cross-sectional study in Zhejiang and Henan Provinces, we found that the immunization coverage and knowledge of non-NIP vaccines among local urban children and caregivers was highest, followed by migrant, non-left-behind and left-behind children and caregivers. The satisfaction rate of non-NIP vaccination was quite high (> 90%) among the caregivers of four family types, and those from left-behind families were more satisfied with non-NIP vaccination compared to local urban children. The immunization coverage of non-NIP vaccines was associated with characteristics of children and caregivers, including age of children, and family role, age, sex, physical health and non-NIP vaccination knowledge of caregivers. The core node of the interrelationship among non-NIP vaccination coverage, knowledge and satisfaction was the satisfaction of vaccination schedule.

Prior research has provided information about the disparities on immunization coverage between rural and urban children [27, 28], while our study focused on the inequality in non-NIP vaccination among two vulnerable groups of migrant and left-behind children and their counterparts. Our findings were supported by a previous qualitative study of immunization providers in Sichuan, Guangdong and Henan provinces in China, which revealed the low vaccination coverage in migrant and left-behind children [29]. Compared to the caregivers from local urban families, we found those from migrant, non-left-behind and left-behind families showed significantly lower knowledge on non-NIP vaccination. Caregivers from migrant families and rural families may have lower socio-economic status, relatively scarce sources of information [30, 31], and thus have poorer understanding on non-NIP vaccination and less access to healthcare utilization. To improve vaccination service quality and reduce rural-urban health inequalities, children from migrant and left-behind families should be prioritized in the non-NIP vaccines promotion programs. Vaccination education could also have the potential to improve the caregivers’ knowledge and acceptance on non-NIP vaccines, strengthen their connection with local vaccinators, and treat non-NIP vaccination correctly. However, the caregivers from left-behind families in our study showed highest satisfaction on non-NIP vaccination. The caregivers from left-behind families were mainly grandparents, who lived in rural communities for decades, and tended to have a harmonious relationship with community vaccinators [32]. Results from the coverage-knowledge-satisfaction network models of non-NIP vaccination suggesting optimization on vaccination schedule would contribute to caregivers’ satisfaction on non-NIP vaccination for their children, and thus improve immunization coverage of non-NIP vaccines.

The association between socio-demographic characteristics of families and immunization coverage of non-NIP vaccines have been suggested in previous observational studies. An online cross-sectional survey conducted in Jiangsu Province of China suggested parents with younger age, lower education level and health-related occupations had lower acceptance on non-NIP vaccines for their children [33]. As suggested by a recent qualitative study, the child-related determinants of low immunization coverage included sex, siblings and health conditions, while the caregiver-related determinants included socio-economic status, family role, education level and ethnicity [29]. In our study, children aged > 2 years were more likely to receive all five non-NIP vaccines among most types of families. It might be because older children were physically stronger than younger ones, and had less contradictions for vaccination [34]. In addition, some caregivers might have poor access to the vaccination information, especially those from migrant and rural families, and non-NIP vaccinations would be given as the child grows older [34]. Caregivers with better physical health and younger ages would have higher autonomy and energy to get their children received non-NIP vaccines, while caregivers being parents or females, who tended to be more concerned about vaccine adverse events, showed lower probability to take their children for non-NIP vaccination [35]. These findings indicated non-NIP vaccination promotion should be delivered early for families with age-eligible children, and younger family members should be encouraged to participate in the decision-making process of non-NIP vaccination. The opposite association between immunization knowledge level and coverage was observed between local urban families and left-behind families, which might be explained by the difference in sources of information [36]. Caregivers from local urban families had more sources to receive information of vaccination, and the information could be mixed and biased, leading caregivers to express more concerns on non-NIP vaccination [37]. On the contrary, caregivers from left-behind families, mainly grandparents, had more satisfaction with local vaccination services as mentioned above, and therefore were more willing to take their children for non-NIP vaccination. Our findings add weight to the evidence that the effects of child-related factors (e.g., age) and family structure and socio-economic status on the caregiver’s decision-making process on non-NIP vaccination should be stressed. To inform broadly applicable strategies for promoting non-NIP vaccination among migrant and left-behind children, the government should add more vaccines to publicly funded immunization schedule or make policy to decrease the price of non-NIP vaccines to an affordable extent.

Strengths of our study include the consideration of families of four migration types in one study design and the use of network models. In addition, our study had no missing data of variables, since caregivers were incentivized to participate in the survey by providing reasonable money. Limitations to this study also warrant consideration. First, the cross-sectional design precluded us to capture the dynamic change of immunization coverage, knowledge and satisfaction, and to explore the reason of low immunization coverage in migrant and left-behind children. Second, the survey was conducted in local township health centers and community health service centers, which could cause selection bias. For example, the vulnerable groups, migrant families and left-behind families were less accessible to the local vaccine clinics, leading to the overestimate of non-NIP vaccines coverage in these population. Third, recall bias and social desirability bias might exist, and the latter referred when respondents give answers to questions that they believe will make them look good to others, concealing their true opinions or experiences [38]. For example, caregivers might report higher satisfaction with non-NIP vaccination. Fourth, our study only included five non-NIP vaccines (Hib vaccine, varicella vaccine, rotavirus vaccine, EV71 and PCV13), and other common non-NIP vaccines, such us influenza vaccine, were not considered. Fifth, due to the small sample size, the sample of some categories of variables were too small (e.g., nationality of children and caregivers, and the occupation of caregivers) to be included in the models. The small sample size also limited the further network analyses among families of four types. Future large studies are warranted to evaluate more associated factors of non-NIP vaccines coverage and the interplay of immunization coverage, knowledge and satisfaction of non-NIP vaccines among migrant and left-behind families. Sixth, we did not assess NIP vaccination as comparators, and whether the immunization disparities among migrant and left-behind families were specific to non-NIP vaccines or general to both NIP and non-NIP vaccines remained unclear. Finally, the sex of children and caregivers only included two categories of males and females, and “intersex” was not included. In addition, some important information of non-NIP vaccines, including the price and accessibility, was not surveyed. However, we investigated the effects of socioeconomic status of families on non-NIP vaccination, also providing support for lowering the prices of non-NIP vaccines in the future.

## Conclusions

The disparities of immunization coverage and knowledge of non-NIP vaccines existed among migrant families in urban areas, and non-left-behind and left behind families in rural areas. Age of children, physical health of caregivers and family structures were associated with the immunization coverage of non-NIP vaccines. When attempting to reduce health inequality in non-NIP vaccination for the children, attention should be paid not only the rural–urban disparity but also the vulnerable groups of left-behind and migrant children.

### Supplementary Information

**Additional file 1:** Questionnaire. immunization coverage, knowledge and satisfaction of non-NIP vaccines.

**Additional file 2:** Table. Checklist for Reporting Of Survey Studies (CROSS).

## Data Availability

Data and materials are available from the authors upon reasonable request.

## Abbreviations

NIP: National Immunization Program
Hib: Hemophilus influenza b
EV71: Enterovirus 71 vaccine
PCV13: 13-Valent pneumonia vaccine
*PR*: Prevalence ratio
*OR*: Odds ratio
*CI*: Confidence interval
SF-12: 12-Item short form survey
*SD*: Standard deviation
ANOVA: One-way analysis of variance
MGM: Mixed graphical model
LASSO: Least absolute shrinkage and selection operator
EBIC: Extended Bayesian Information Criterion
SC: Stability coefficient
