# Supplementary material for: Immunization coverage, knowledge, satisfaction, and associated factors of non-National Immunization Program vaccines among migrant and left-behind families in China: evidence from Zhejiang and Henan provinces
Source: Infect Dis Poverty. 2023 Oct 13;12:93. doi: 10.1186/s40249-023-01145-5 (PMC10571434; doi:10.1186/s40249-023-01145-5)
Supplement: Supplementary file 1 — Additional file 1: Questionnaire. immunization coverage, knowledge and satisfaction of non-NIP vaccines. [file 40249_2023_1145_MOESM1_ESM.docx]

**Questionnaire. immunization coverage, knowledge and satisfaction of non-NIP vaccines**

**Section A: Characteristics of children**

A1. Name:

A2. Nationality:

A3. Sex:

(i) Male

(ii) Female

A4. Date of birth:

A5. Hukou status:

(i) Rural

(ii) Urban

(iii) None

A6. Has the interviewed child been living with their parents from birth to the present?

(i) Yes (skip to A9)

(ii) No

A7. Which parent(s) were not living with the interviewed child?

(i) Father

(ii) Mother

(iii) Both father and mother

A8. How long has the parent(s) been living with the interviewed child?

DD/MM/YYYY to DD/MM/YYYY

A9. Where is the hukou of the interviewed child?

A10. Where is the interviewed child living now? How long?

A11. Which family members are living with the interviewed child?

(i) Father

(ii) Mother

(iii) Grandfather

(iv) Grandmother

(v) Siblings

A12. How many family members aged < 6 years?

A13. How many siblings does the interviewed child have? What is the birth order among the child’s siblings?

**Section B: Characteristics of caregivers**

B1. What is your relationship with the interviewed child?

(i) Father

(ii) Mother

(iii) Grandfather

(iv) Grandmother

(v) Others

B2. Age:

B3. Sex:

(i) Male

(ii) Female

B4. Nationality:

B5. Hukou status:

B6. Education level:

(i) Illiteracy

(ii) Primary school

(iii) Junior high school

(iv) Senior high school/vocational school/technical secondary school

(v) Junior college

(vi) Bachelor's degree or higher

B7. Occupation:

(i) Retired

(ii) Farmer

(iii) Teacher or researcher

(iv) Health worker

(v) Civil servant

(vi) Business and service employee

(vii) Individual household

(viii) Enterprise employee

(ix) Worker

(x) Student

(xi) Housewife

(xii) Unemployed

(xiii) Other

B8. How much was your total household income (including earning income, capital income, pension income, income from government transfers, other income and the total income from other household members) last year?

B9. Where is your hukou?

B10. Where are you living now? How long?

B11. Please choose your answer according to your actual situation.

B11.1. In general would you say your health is:

(i) Excellent

(ii) Very good

(iii) Good

(iv) Fair

(v) Poor

B11.1a. Were you suffering from any long-term chronic conditions last year?

(i) Yes

(ii) No (skip to B11.2)

B11.1b. Does the chronic conditions limit you in daily activity?

(i) Yes

(ii) No

The following questions are about activities you might do during a typical day. Does your health now limit you in these activities? If so, how much?

B11.2. Moderate activities, such as moving a table, pushing a vacuum clearner, bowling, or playing golf.

(i) Yes, limited a lot

(ii) Yes, limited a little

(iii) No, not limited at all

B11.3. Climbing several flights of stairs

(i) Yes, limited a lot

(ii) Yes, limited a little

(iii) No, not limited at all

During the past week, have you had any of the following problems with your work or other regular daily activities as a result of your physical health?

B11.4. Accomplished less than you would like

(i) Yes

(ii) No

B11.5. Accomplished less than you would like

(i) Yes

(ii) No

During the past week, have you had any of the following problems with your work or other regular daily activities as a result of any emotional problems (such as feeling depressed or anxious)?

B11.6. Accomplished less than you would like

(i) Yes

(ii) No

B11.7. Accomplished less than you would like

(i) Yes

(ii) No

B11.8. During the past week, how much did pain interfere with your normal work (including both work outside the home and housework)?

(i) Not at all

(ii) A little bit

(iii) Moderately

(iv) Quite a bit

(v) Extremely

These questions are about how you feel and how things have been with you during the past week. For each question, please give the one answer that comes closest to the way you have been feeling. How much of the time during the past week

B11.9. Have you felt calm and peaceful?

(i) All of the time

(ii) Most of the time

(iii) A good bit of the time

(iv) Some of the time

(v) A little of the time

(vi) None of the time

B11.10. Did you have a lot of energy?

(i) All of the time

(ii) Most of the time

(iii) A good bit of the time

(iv) Some of the time

(v) A little of the time

(vi) None of the time

B11.11. Have you felt downhearted and blue?

(i) All of the time

(ii) Most of the time

(iii) A good bit of the time

(iv) Some of the time

(v) A little of the time

(vi) None of the time

B11.12. During the past week, how much of the time has your physical health or emotional problems interfered with your social activities (like visiting with friends, relatives, etc.)?

(i) All of the time

(ii) Most of the time

(iii) Some of the time

(iv) A little of the time

(v) None of the time

**Section C. Immunization coverage of children**

C1. Has your child received any of the following non-NIP vaccines?

C1a. Haemophiles influenza b (Hib) vaccine

(i) Yes

(ii) No

C1b. Varicella vaccine

(i) Yes

(ii) No

C1c. Rotavirus vaccine

(i) Yes

(ii) No

C1d. Enterovirus 71 vaccine (EV71)

(i) Yes

(ii) No

C1e. 13-valent pneumonia vaccine (PCV 13)

(i) Yes

(ii) No

C2. What is your reason for not taking your child for vaccination?

(i) Never heard this vaccine

(ii) Illness of the child

(iii) No risk of contacting the disease

(iv) Bad experience of vaccination

(v) Received the same type

(vi) Concern about safety

(vii) Concern about efficiency

(viii) Too many doses to vaccinate

(ix) Insufficient supply of vaccines

(x) Don’t know the place

(xi) Limited service time

(xii) Heard of negatice information

(xiii) Others don’t suggest

(xiv) Having no time

C3. Would you like to vaccinate your children against the following vaccines in the future

C3a. Haemophiles influenza b (Hib) vaccine

(i) Yes

(ii) No

C3b. Varicella vaccine

(i) Yes

(ii) No

C3c. Rotavirus vaccine

(i) Yes

(ii) No

C3d. Enterovirus 71 vaccine (EV71)

(i) Yes

(ii) No

C3e. 13-valent pneumonia vaccine (PCV 13)

(i) Yes

(ii) No

**Section D:** **Immunization knowledge of caregivers**

Please select the option that you think is correct.

Convenience

D1. The child could accept vaccination nationwide as long as he/she had a vaccination certificate

(i) Yes

(ii) No

(iii) Unknown

Category

D2. In China, vaccines are divided into National Immunization Program (NIP, Category A) vaccines and non- National Immunization Program (non-NIP, Category B) vaccines.

(i) Yes

(ii) No

(iii) Unknown

Efficiency

D3. Vaccination is the most effective way to prevent infectious diseases.

(i) Yes

(ii) No

(iii) Unknown

Continuity

D4. The vaccine gives lifelong immunity.

(i) Yes

(ii) No

(iii) Unknown

Time

D5. Vaccination of the child should be strict to the plan.

(i) Yes

(ii) No

(iii) Unknown

Schedule

D6. The caregiver should inform the vaccinator about the health of the child before vaccination.

(i) Yes

(ii) No

(iii) Unknown

D7. The child should be kept in the vaccination clinic or observation room for at least 30 minutes after vaccination.

(i) Yes

(ii) No

(iii) Unknown

D8. According to the health status of the child, vaccination may not be recommended or may be postponed.

(i) Yes

(ii) No

(iii) Unknown

Adverse events

D9. It is normal for the child to have a fever below 38 °C after vaccination.

(i) Yes

(ii) No

(iii) Unknown

D10. The child should seek medical attention immediately if they have ulcers and suppurative infections at the vaccination site after vaccination.

(i) Yes

(ii) No

(iii) Unknown

**Section E. Immunization satisfaction of caregivers**

Please select the option that you think is correct according to your immunization experience.

Convenience

E1. The extent of convenience to the vaccination clinic.

(i) Very satisfied

(ii) Satisfied

(iii) Normal

(iv) Unsatisfied

(v) Very unsatisfied

Vaccination reminder

E2. The way to remind the caregiver to get their child vaccinated.

(i) Very satisfied

(ii) Satisfied

(iii) Normal

(iv) Unsatisfied

(v) Very unsatisfied

Vaccination environment

E3. The environment of the vaccination clinic

(i) Very satisfied

(ii) Satisfied

(iii) Normal

(iv) Unsatisfied

(v) Very unsatisfied

E4. The sanitation status of the vaccination

(i) Very satisfied

(ii) Satisfied

(iii) Normal

(iv) Unsatisfied

(v) Very unsatisfied

Consultation

E5. Vaccinators’ answers for the questions of the caregiver.

(i) Very satisfied

(ii) Satisfied

(iii) Normal

(iv) Unsatisfied

(v) Very unsatisfied

E6. Enquiry of vaccinators about health status of the child before vaccination.

(i) Very satisfied

(ii) Satisfied

(iii) Normal

(iv) Unsatisfied

(v) Very unsatisfied

E7. Notification of the vaccination for this time to the caregiver.

(i) Very satisfied

(ii) Satisfied

(iii) Normal

(iv) Unsatisfied

(v) Very unsatisfied

E8. Notification of the vaccination for next time to the caregiver.

(i) Very satisfied

(ii) Satisfied

(iii) Normal

(iv) Unsatisfied

(v) Very unsatisfied

E9. Notification of do’s and don’ts of vaccination to the caregiver.

(i) Very satisfied

(ii) Satisfied

(iii) Normal

(iv) Unsatisfied

(v) Very unsatisfied

Vaccination skills

E10. The vaccination skills of vaccinators.

(i) Very satisfied

(ii) Satisfied

(iii) Normal

(iv) Unsatisfied

(v) Very unsatisfied

Service quality

E11. The service attitude of vaccinators.

(i) Very satisfied

(ii) Satisfied

(iii) Normal

(iv) Unsatisfied

(v) Very unsatisfied

Vaccination process

E12. The arrangement of vaccination process.

(i) Very satisfied

(ii) Satisfied

(iii) Normal

(iv) Unsatisfied

(v) Very unsatisfied

Vaccination education

E13. The publicity and education of vaccination.

(i) Very satisfied

(ii) Satisfied

(iii) Normal

(iv) Unsatisfied

(v) Very unsatisfied

Time of vaccination

E14. The arrangement of time of vaccination.

(i) Very satisfied

(ii) Satisfied

(iii) Normal

(iv) Unsatisfied

(v) Very unsatisfied
